# Ellipsometry Study of CdSe Thin Films Deposited by PLD on ITO Coated Glass Substrates

**DOI:** 10.3390/ma14123307

**Published:** 2021-06-15

**Authors:** Flavia P. N. Inbanathan, Pawan Kumar, Kiran Dasari, Ram S. Katiyar, Jixin Chen, Wojciech M. Jadwisienczak

**Affiliations:** 1 School of Electrical Engineering and Computer Science, Ohio University, Athens, OH 45701, USA; jadwisie@ohio.edu; 2 Department of Physics, Gurukula Kangri Vishwavidyalaya, Haridwar, Uttarakand 249404, India; pksoniyal13@gmail.com; 3 Department of Physics, University of Puerto Rico, San Juan, PR 00936, USA; ram.katiyar@upr.edu; 4 Department of Chemistry and Biochemistry, Ohio University, Athens, OH 45701, USA; chenj@ohio.edu

**Keywords:** CdSe, PLD, XRD, AFM, absorbance, Tauc plot, band gap, ellipsometry

## Abstract

Cadmium selenide (CdSe) thin films were deposited on indium tin oxide (ITO) coated glass substrates using pulsed laser deposition (PLD) technique under different growth temperatures. Samples were investigated for their structural, morphological, and optical properties through X-ray diffraction (XRD), atomic force microscopy (AFM), and UV-Vis-NIR spectroscopy. AFM analysis revealed that the surface roughness of the as-grown CdSe thin films increased with the increase in deposition temperature. The optical constants and film thickness were obtained from spectroscopic ellipsometry analysis and are discussed in detail. The optical band gap of the as-grown CdSe thin films, calculated from the Tauc plot analysis, matched with the ellipsometry measurements, with a band gap of ~1.71 eV for a growth temperature range of 150 °C to 400 °C. The CdSe thin films were found to have a refractive index of ~3.0 and extinction coefficient of ~1.0, making it a suitable candidate for photovoltaics.

## 1. Introduction

The spectroscopic ellipsometry technique is a useful tool for revealing the electronic structure and complex optical properties of semiconductors by measuring the optical responses of a flat substrate to polarized light. It is mainly used to measure film thickness and the optical constants. For novel photoelectronic device design, knowing the optical constants such as the refractive index and dielectric constant is essential for studying the pattern of light being guided through the thin films [1]. Group II-VI compound semiconductors and their alloys, with a band gap energy ranging from 1.20 eV to 3.91 eV, have been explored for their electronic and photonic applications [2,3,4]. In result of this, to date, different band gap energies for binary and more complex alloys have been achieved via proper band gap engineering approaches. It was demonstrated that the II-VI materials’ optical response can be varied from infrared to the ultra-violet region, which, among others, paved the way for solar cell applications. In particular, the cadmium selenide (CdSe) binary metal chalcogenides occur as an n-type semiconductor with a direct band gap of 1.73 eV [2,3,4]. They also have a high absorption coefficient of *α* = 10^4^ cm^−1^ at 720 nm [1]. For this reason, a much thinner CdSe film is sufficient to effectively absorb the sunlight, compared with other II-VI contemporary photovoltaic materials. On the other hand, CdSe has interesting size controlled optical and electrical properties. Owing to these it has been widely explored in the recent past for its suitability for various applications, including light emitting diodes [5], solid-state solar cells [5], photoconductors [6,7], photovoltaic cells [8], photoelectrochemical cells [9], and sensors [10,11]. It is known that the band gap of ternary and quaternary semiconductor alloys is tunable based on lattice constants engineering and the size of the particles, due to the quantum confinement size effect. So far, various II-VI compound materials, such as CdTe(_1−y_)S_y_ and CdTe_(1−x)_Se_x_, etc., have been tested to replace CdS layers [12]. It was found that CdSe thin film can be an alternative n-type layer replacing CdS layer in solar cell devices. Furthermore, it was shown that graded alloy compounds such as CdSe_1-x_Te_x_ can be formed due to the interdiffusion between the CdSe and CdTe layers. Nevertheless, CdSe holds distinct advantages over CdS, such as a smaller lattice mismatch between CdSe and CdTe, as compared to the CdS and CdTe counterpart [12,13], which makes it critical for improving the solar cell short circuit current density (*J_SC_*), due to the improved quality of the interfaces between layers and grain boundaries, as well as the increased optical absorption compared to a CdS layer. Moreover, it was found from spectroscopic ellipsometry mapping analysis of the CdS/CdSe/CdTe graded layers processed at elevated temperature that combining CdSe and CdTe thin films led to better conversion of photo-inactive CdSe to photo-active CdTe_(1−x)_Se_x_, which in turn resulted in a photocurrent increase, reduction of the short wavelength losses, and improved collection of photons at longer wavelengths, respectively [12]. Various deposition techniques, such as thermal evaporation [6], sputtering [8], pulsed laser deposition [14,15], chemical vapor deposition [16], chemical bath deposition [17], spray-pyrolysis [18], electrodeposition [19,20], and molecular beam epitaxy (MBE) [21], have been well explored to fabricate high quality II-VI thin films. Among all the deposition techniques mentioned, PLD can produce a thin film without changing the chemical composition, as the layer is formed by vaporizing the target material with a laser beam, similar to the MBE growth process. Moreover, the stability of the CdSe material is a prominent issue for applications, as an increase in growth temperature leads to surface roughness and oxide layer formation, affecting the optical properties [22]. Hence, a suitable deposition method such as PLD can successfully counter these issues. PLD is known to be a layer-by-layer growth technique, as evident from the time-resolved surface X-ray diffraction (XRD) measurements showing that crystallization and the majority of interlayer mass transport occur on time scales that are comparable to those of the plume/substrate interaction [23]. Furthermore, PLD is a vapor phase deposition process, during which the growth of materials from a PLD plume is fundamentally different than those found in thermal evaporation. With the advancements in PLD process development with respect to improved control mechanism over thin film deposition parameters and resulting film quality, the term “laser-MBE” was coined to describe a PLD layer-by-layer growth method where RHEED (reflection high energy electron diffraction) is involved [24]. Furthermore, PLD has already been successfully employed to deposit a variety of technologically relevant materials [14,16]. In principle, PLD is a thin-film deposition technique employing high-energy laser pulses to vaporize the surface of a solid target in a vacuum setting, with subsequent condensing of the vapor on a substrate to form a thin film of a well-controlled thickness. Thus, the main benefit of PLD derives from the laser induced material evaporation mechanism, based on rapid explosion of the target surface region due to superheating induced by photon(s) absorption by the target. Furthermore, PLD is an easily adoptable method, with well-defined control over the high growth rate, stoichiometric transfer, unrestricted degree of freedom in the ablation geometry compared to other deposition techniques, flexibility in the used laser wavelength and power density, and the ability to deposit multiple layers. Unlike other thermally driven evaporation physical deposition methods, relying on producing a vapor composition dependent on the vapor pressures of the elements in the target material, the PLD removal produces a stoichiometric plume of material akin to that of the target. Hence, it is easier to obtain, compared to alternative deposition techniques, the required film stoichiometry, especially when multielement materials are involved. It is also known that the kinetic energies of ablated particles are insufficient to induce bulk damage; however, they are high enough to stimulate the surface diffusion desired for good quality film growth. Hence, PLD has been recognized as being suitable for a wide range of material deposition scenarios where excellent film adhesion is required. Furthermore, a high cooling rate during PLD promotes disordered film formation, similarly to nanocrystalline or amorphous films. Since PLD offers good controllability of the film microstructure, by properly selecting the laser operating conditions, one can foresee a facile manufacturing process of II-VI semiconductors for PV applications by better controlling of the grain size distribution and grain boundary [14]. However, not much literature has reported on the profound analysis of CdSe thin films grown by PLD at different growth temperatures. This work is focused on understanding the non-linear properties of CdSe through a spectroscopic ellipsometry study. The effect of different in situ growth temperatures were studied in terms of structural, morphological, and optical properties, through X-ray diffraction, atomic force microscopy (AFM), and ellipsometry.

## 2. Materials and Methods

Bare glass substrates are nonconducting and hence indium tin oxides (ITO) deposited on glass substrates were used, as ITO acts as a potential bottom electrode for photovoltaic applications. The commercially available ITO coated glass substrates (referred as ITO/glass substrates in further discussion) were purchased from MTI corporation, CA, USA. The thickness of the ITO was ~90 nm ± 10 nm with a resistivity of 16–19 ohm/sq., and with the optical transparency >85% at 555 nm, respectively. The CdSe target was prepared with the source material CdSe from Sigma Aldrich with purity 99.999% and sintered at 850 °C for 12 h in the presence of Argon gas at atmospheric pressure. The CdSe thin films were deposited on ITO/glass substrates under different growth temperatures using a coherent KrF excimer pulsed laser deposition method (Lambda Physik COMPex −205i, λ = 248 nm, 10 ns, 10 Hz). The laser beam was focused onto the target surface, by using a lens with a focal length of 50 cm. The target was kept in a non-stop horizontal and vertical displacement to refresh the ablated zone. The sintered target was placed at 5-cm distance from the substrate holder and was irradiated by the pulsed laser for 10 s to remove the oxides and other contaminations from the target surface prior to the growth cycle. The surface of the substrates was subsequently cleaned with isopropyl alcohol, methanol, and deionized water in an ultrasonic bath before loading them in to the PLD chamber for the growth of the CdSe thin films. A typical KrF excimer laser with wavelength of 248 nm and pulse width of 10 ns and energy of 90 mJ operating at 10 Hz was used. A laser beam of fluence 2.2 J/cm^2^ was introduced into the growth chamber with a repetition rate of 10 Hz and focused onto the target on a 4 mm × 2 mm spot. No reactive gas was introduced into the chamber during the growth. The rotation speed of the target and the substrate holder was fixed at 5 rpm. The CdSe thin films were deposited at different substrate temperatures, ranging from 150 °C to 400 °C, with 50 °C intervals. A resistive type heater was used to heat the substrate temperature to arrive at the set value. Laser ablation was done inside the vacuum chamber, which was kept at 10^−6^ Torr. The growth pressure was maintained at 1.5 × 10^−6^ Torr, whereas the base pressure was kept at 8 × 10^−8^ Torr by utilizing a molecular pump along with a mechanical pump. The films with thickness of about 200 nm were targeted using the above method. The crystalline nature of the as-grown films was characterized by X-ray diffraction (Rigaku Smartlab-XRD, Rigaku corporation, Tokyo, Japan) using copper K-α radiation (*λ* = 1.5406 Å). The elemental analysis of the as-grown thin films was studied by X-ray photoelectron spectroscopy (Physical Electronics, PHI-5600 XPS). The surface morphology and the roughness of the films were investigated by atomic force microscopy (Multi-mode Nanoscope V 7.30r, Veeco Instruments Inc., Santa Barbara, CA, USA). The absorbance was measured using UV-Vis-NIR spectroscopy using an Agilent HP-8453 diode-array spectrophotometer, with a spectral range from 190 nm to 1100 nm, and the optical band gap was calculated using the Tauc plot method. The thickness of the films and the dielectric constants were analyzed using a Horiba Uvisel ellipsometer (Horiba, NJ, USA) for selected CdSe thin films deposited on ITO/glass substrate at 150, 250, 300, and 400 °C at an incident angle of 70° in the spectral range of 250 nm to 900 nm, with an increment of 2 nm, respectively.

## 3. Results and Discussion

### 3.1. Crystallinity

**Figure 1:**
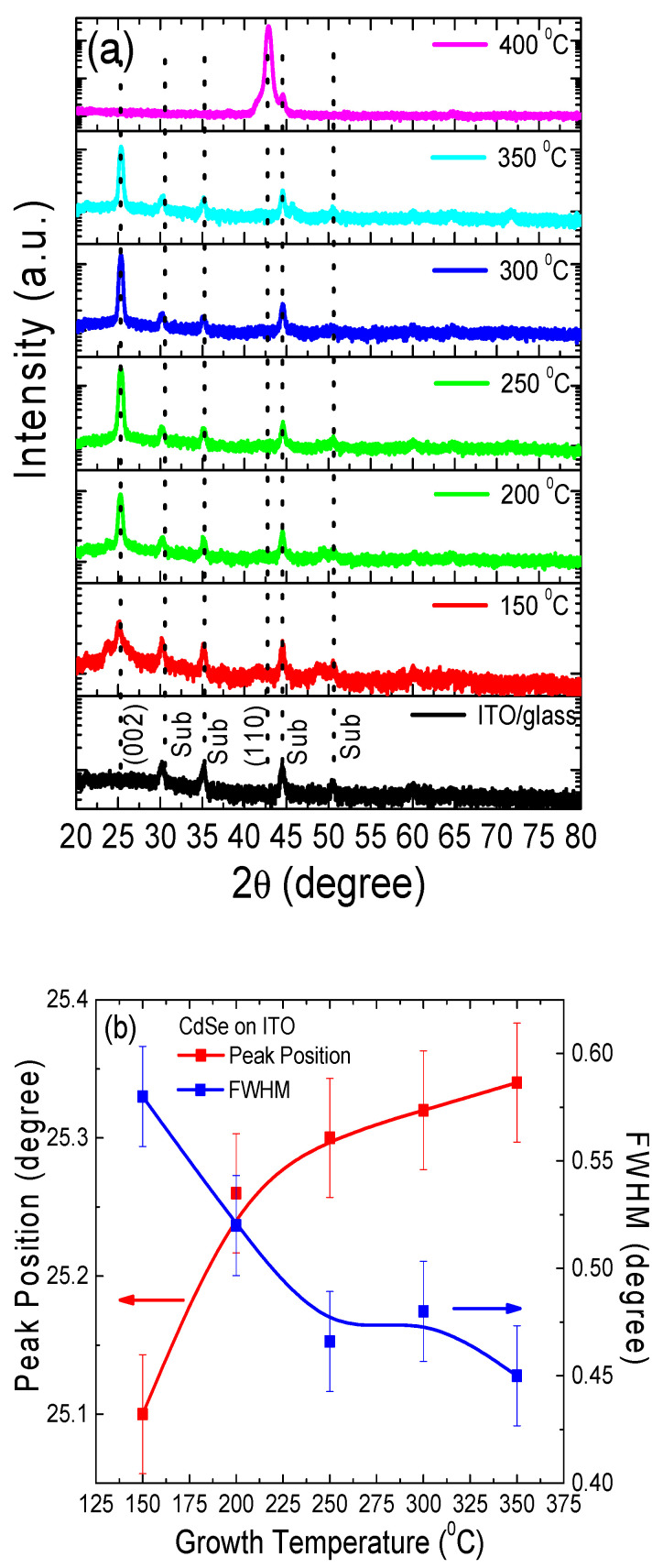
(**a**) XRD patterns of the CdSe thin films showing a wurtzite phase with a peak at (002) for samples deposited at 150 °C to 350 °C, and peak (110) for the sample deposited at 400 °C. (**b**) Growth temperature dependent XRD (002) peak position and its FWHM change for the CdSe thin films. The solid lines are a guide for the eyes.

The XRD patterns of the films deposited at the substrate temperatures from 150 °C to 400 °C, along with the peak position and full width half maxima (FWHM) are shown in Figure 1a,b. It is known that CdSe can crystallize in cubic (zinc-blende type) or hexagonal (wurtzite type) structures; however, a cubic structure is a metastable phase whereas a hexagonal is a stable phase. XRD analysis confirmed the crystallinity of all the PLD films grown under different growth conditions. A highly oriented CdSe (002) peak at 25.3° was observed without indication of any secondary phase in all the samples, except at 400 °C. The XRD analysis indicated that the films had a single crystalline hexagonal structure (JCPDS file:00-002-0330) with the space group P3mc [25]. The decrease in the FWHM (0.58° to 0.45°) of (002) peak of the CdSe thin films with the increase in growth temperature confirmed the enhancement in the crystallinity of the films. The XRD spectrum for the CdSe sample deposited at 400 °C was dominated by a crystal orientation (110) peak corresponding to hexagonal phase, as typically observed in chalcogenide film grown at higher temperatures. The observed change in sample morphology was further corroborated by the ellipsometry spectra (see Section 3.4), as evidenced by the lack of well-defined absorption edge peak in the 400 °C sample, observed for the other CdSe samples deposited at lower temperatures. The CdSe film crystallite size was estimated by applying Scherrer’s formula, as given in Equation (1),

(1) $$D=\frac{k\lambda }{\beta \cos\theta }$$

where *k* is the shape factor (0.94 in our case), *λ* is the X-ray wavelength, *β* is the FWHM radians, and *Θ* is the Bragg’s angle. The typical shape factor varies from 0.89 to 1.39. In addition to the enhancement in the FWHM, the crystallite size was also found to increase from 14.76 nm (150 °C) to 18.9 nm (350 °C).

### 3.2. Morphology Analysis

**Figure 2:**
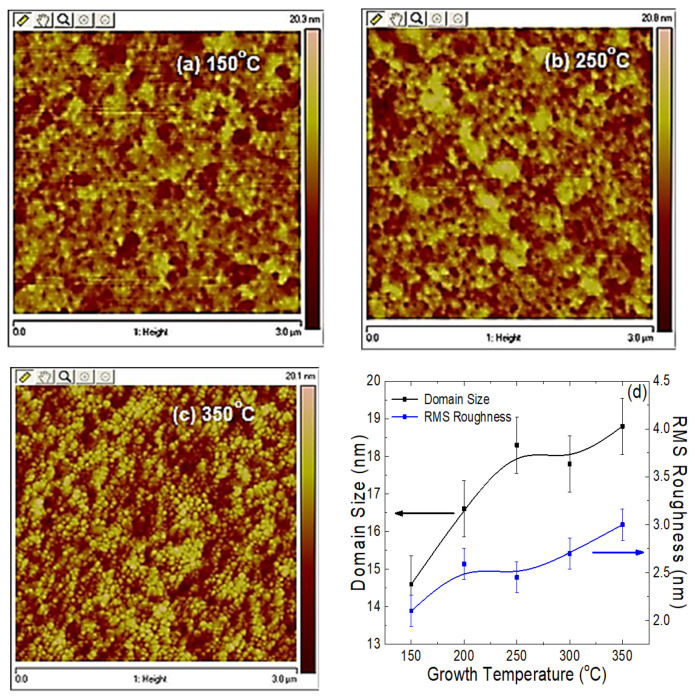
AFM images of the CdSe thin films surface grown at (**a**) 150 °C, (**b**) 250 °C, and (**c**) 350 °C. (**d**) Growth temperature dependent grain size and surface roughness analysis for the CdSe thin films deposited at different growth temperatures. Solid lines are a guide for the eyes.

The surface morphology of the as-deposited films was measured in AFM contact mode and a root mean square (RMS) roughness analysis was performed, as shown in Figure 2a–c. As expected from the PLD method, the surface of all the films was found to be comparatively smooth, irrespective of the growth temperature (Figure 2d). The films grown at lower temperatures showed a smoother surface compared to the films grown at higher temperature. It is known that the film roughness depends strongly on the grain and grain-boundary (e.g., domain) size, which are affected by growth temperature.

Thus, typically, these two parameters obtained from the AFM and XRD closely matched with each other. Figure 2d shows an increase of RMS roughness for the sample grown at 200 °C, which was an unanticipated deviation from the otherwise expected linear trend. The specific reason for this anomaly is unclear at the present. As shown in Table 1, the surface roughness of the CdSe films deposited by PLD was smallest for the material deposited at 150 °C and changed nonmonotonically, with a clearly increasing trend.

### 3.3. Optical Analysis

**Figure 3:**
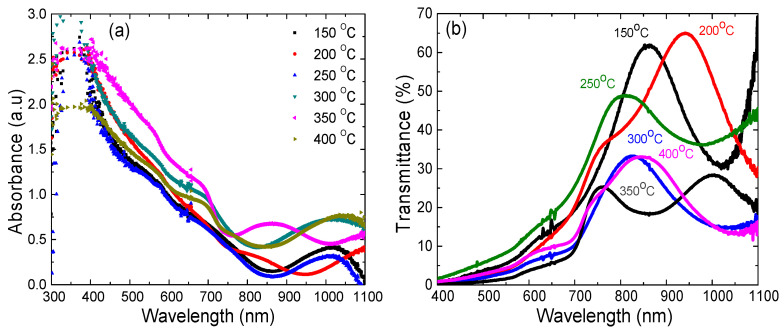
(**a**) Absorbance vs. wavelength and (**b**) transmittance vs. wavelength for CdSe thin films deposited on ITO/glass substrates at different temperatures.

First, the optical properties of CdSe thin films grown on ITO/glass substrate were studied through absorbance and transmittance. Both the absorbance and the complementary transmittance data are shown in Figure 3a,b. An absorbance decrease (>80%) within the solar absorption window 400–700 nm was observed for all CdSe samples, as shown in Figure 3a. The interference fringes observed from 800 nm to 1100 nm were due to the constructive and destructive interference of the wave fronts from the CdSe/ITO interface and the top layer of the CdSe surface open to the air, consistent with the literature [19]. The lower absorbance edge in the NIR spectral region for the samples grown at 350 °C and 400 °C compared to the other samples deposited at lower temperatures, we believe was due to an increase in the domain size that was confirmed in AFM analysis (see Figure 2d). Furthermore, the CdSe band gap energy was reduced with the increase of growth temperature (see Table 2). This argument is supported further by the fact that the interatomic spacing increased when the amplitude of the atomic vibrations increased, due to the thermal energy [26]. This in turn decreased the potential seen by the electrons in the material, which reduced the energy band gap.

The practical aspect of optical transmittance spectra, when considering photovoltaics, is primarily demonstrated in the spectral region below the CdSe band gap. Figure 3b shows that the peak transmittance below the band gap of CdSe (1.71 eV) was higher for the samples grown at temperatures 150–250 °C (50–65%) than those grown at 300–400 °C (20–30%). We believe that the variations in both absorbance and transmittance spectra were mainly due to the CdSe thin film thickness non-uniformity, light interference phenomenon, domain size distribution, and variation in CdSe grain packing density.

**Figure 4:**
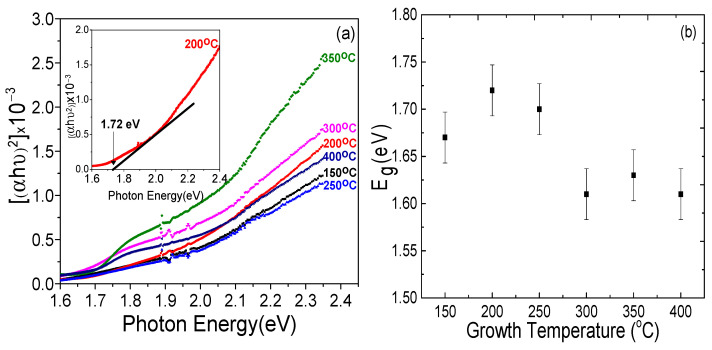
(**a**) Tauc plot of CdSe thin films grown at different temperatures from 150 °C to 400 °C. Insert shows the example of fitting outcome for CdSe sample grown at 200 °C. (**b**) Growth temperature vs. *E_g_* band gap energy obtained from (**a**).

The band gap of the CdSe thin films studied were determined from absorption spectra measured in UV-Vis-NIR range using Beer Lambert’s law, as shown in Figure 3a. The Tauc plot analysis for the obtained results is shown in Figure 4a [26]. The band gap energy was determined by using Equation (2) applicable to near edge optical absorption of a CdSe semiconductor,

*αhν* = *K*(*hν* −* E_g_*)^*n*^ (2)

where α is the absorption coefficient, *hν* is the photon energy, *K* is a constant, *E_g_* is the optical band gap energy, and n is a constant of 0.5 for an allowed CdSe direct band semiconductor; whereas *n* = 1.5, 2, 3 for direct forbidden transitions, indirect allowed transitions, and indirect forbidden transitions, respectively. The absorption coefficient α is calculated using Equation (3),

*α* = (2.303 × *A*)/*t* (3)

where *A* is the film absorbance in (a.u.) (i.e., absorbance unit), *t* is film thickness in (nm), and photon energy *hν* is calculated using *hν* = 1239.83/*λ*, where *λ* is the wavelength in (nm).

Figure 4a shows the Tauc plot, i.e., photon energy *hν* on the abscissa and the (*αhν*)^2^ on the coordinate [27]. The linear portion of the curve was extrapolated to the *X*-axis and the optical band gap energy of the corresponding thin films was obtained from the intercept as shown in Figure 4a inset, following the method presented by Makula et al. [28]. Using the Tauc plot approach, the optical band gaps were found to be 1.67 eV (150 °C), 1.72 eV (200 °C), 1.7 eV (250 °C), 1.61 eV (300 °C), 1.63 eV (350 °C), and 1.61 eV (400 °C) for the different growth temperatures. It is seen in Figure 4b that when the growth temperature increased, the band gap energy decreased. Typically, one can expect that the semiconductor band gap decreases with increasing growth temperature [29]. Salem et al. showed for CdSe nanoparticles that an increase in growth temperature from 450 °C to 700 °C resulted in a decrease in the optical band gap [30]. They linked this observation to the CdSe particle size increase, resulting in an increase of the number of atoms forming a particle, which in turn made the valence and conduction electrons interaction stronger with the ions’ core within the nanoparticles, decreasing the band gap. Their assumption was that the attraction of the band electrons to the ions’ core in the particles became stronger due to the increase in the number of atoms entering the formation of a CdSe grain, resulting in grain growth, thus causing the band gap decrease [30].

### 3.4. Ellipsometry Analysis

Spectroscopic ellipsometry is suitable for evaluating the thin film optical properties through a complex dielectric function *ε*, or a complex number comprising the refractive index *n* and the extinction coefficient *κ* of the material, as well as the thickness of the thin films. The parameters for the ellipsometry analysis were extracted using the Drude oscillator dispersion model, adopting the Adachi–New–Forouhi formula (i.e., Tauc–Lorentz) [26,31] for the CdSe layer, and both the classical (i.e., Cauchy and Lorentz) and the Adachi formulas for the ITO layer, respectively [27,31]. In the present case, we fixed the thickness of glass in the ITO/glass substrate as 0.69 mm and the ITO layer thickness as 100 nm, respectively, then the thickness of the CdSe layer was fitted using the DeltaPsi2 software and the best fit was confirmed by the obtained minimum *χ^2^* value of less than 12. The absorption coefficient α and extinction coefficient κ are related by Equation (4),

*α* = (4*πκ*)/*λ* (4)

**Figure 5:**
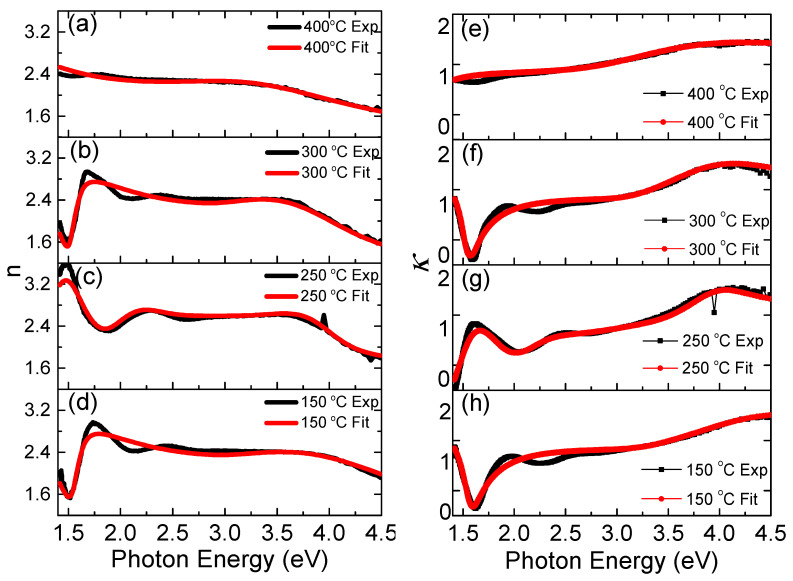
(**a**–**d**) Dependence of refractive index *n* and (**e**–**h**) extinction coefficient *κ* vs. photon energy of CdSe samples grown at different temperatures.

Figure 5 shows the refractive index *n* and extinction coefficient *κ* of the CdSe films deposited at different temperatures extracted from ellipsometry measurements. The estimated value of refractive index was ~2.4 in the visible region (1.7–3.1 eV), which is consistent with the literature [32]. While fitting the experimental data, no significant change was observed in the used dielectric function with the increase in extinction coefficient [32]. This confirmed that the CdSe thin film surface deposited by PLD was nearly uniform, and the surface scattering of the incident light was low. The CdSe thin film deposited at 250 °C showed a maximum refractive index as a resultant of the dielectric contribution of the film and the atomic and molecular oscillations upon light excitation in the photon energy of <1.7 eV. We assume that the refractive index *n* = 3.6 at 1.47 eV, as well as the extinction coefficient maximum value *κ* = 1.2 at the ~1.5 eV, were due to the optimal host crystallinity, domain size, and the presence of excitonic peaks in the near band edge spectral region [33]. The extinction coefficient approached 0 in 1.5–1.7 eV for the CdSe samples grown at 150 °C and 300 °C, whereas it had a value of ~1.0 for samples grown at 250 °C and 400 °C. We inferred that the extinction coefficient of less than 1.0 in the band gap region enabled the electrons located below the band edge to interact with photons, resulting in more absorption when the incident photon energy increased above the band gap energy. This confirmed the high photon absorption with less scattering losses, making it suitable for photovoltaic applications. These results are consistent with the UV-Vis absorption spectra showing that the CdSe thin films were transparent at wavelengths corresponding to photons with energy smaller than the band gap (Figure 3).

**Figure 6:**
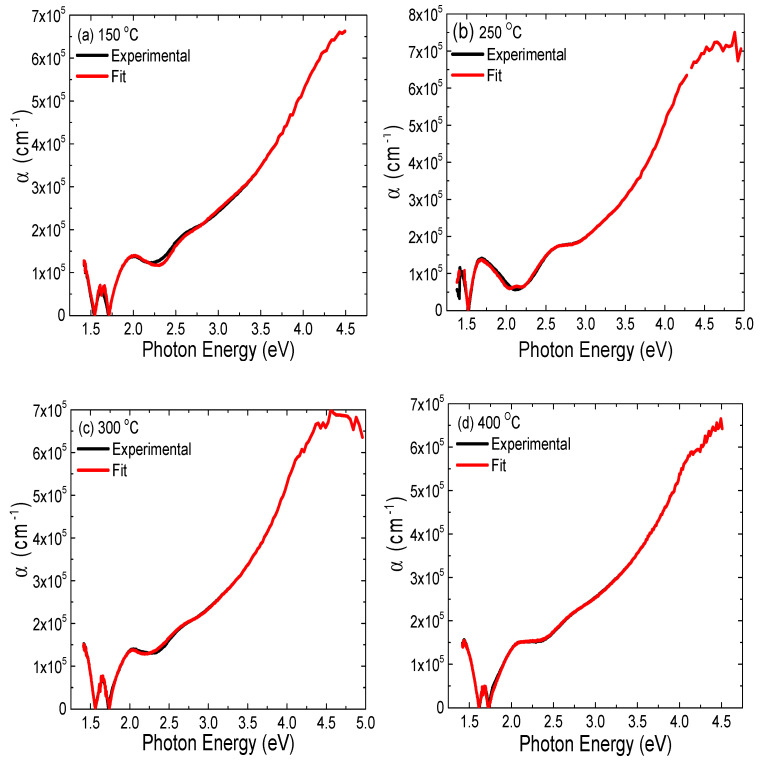
(**a***–***d**) The absorption coefficient *α* vs. photon energy for CdSe thin films deposited at different growth temperatures.

The relationship between the absorption coefficient α and photon energy shown in Figure 6 indicates that major absorbance occurred at the optical band gap energy around ~1.71 eV, due to electron band-to-band transition. This fact reinforces a further benefit of using CdSe as solar light absorbing layer: an expected reduction of the raw material consumption in the photovoltaic device fabrication process [34].

**Figure 7:**
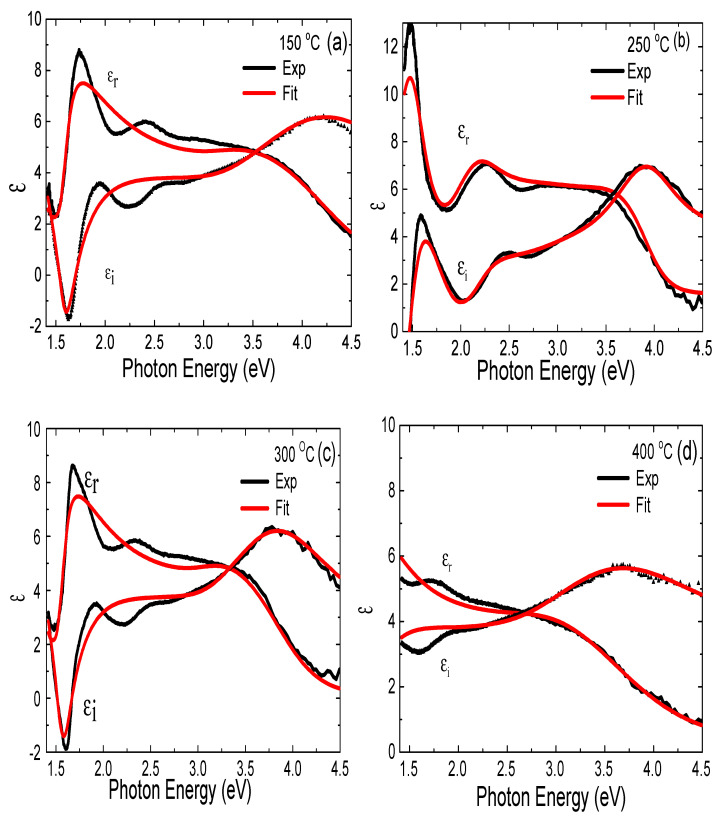
*(***a**–**d**). The dielectric function *ε_r_*, *ε_i_* parameters vs. photon energy of CdSe thin films deposited at different growth temperatures.

Figure 7 shows the dielectric functions *ε_r_*, *ε_i_* parameters determined for the studied CdSe thin films. It was found that there were two excitonic peaks between 1.5 and 2.5 eV for the CdSe thin films grown at 150 °C, 250 °C, and 300 °C, respectively, and only one significant excitonic peak for the CdSe film grown at 400 °C. We accounted for this difference by the CdSe dielectric contribution change and passivation of the film surface states due to higher growth temperature [1,14]. Table 2 shows the CdSe film thickness and energy band gap extracted from the ellipsometry studies. We attributed the observed increase in CdSe film thickness at 250 °C to the fact that the decomposition of the CdSe target on the heated ITO/glass substrate accelerated, due to the thermal activation of the carriers and the deposition of well-adhered CdSe thin films on the ITO/glass substrate. This resulted in a thicker layer than expected (~200 nm). Subsequently, the decrease in the CdSe layer thickness for higher growth temperatures (400 °C) was due to the reduction in deposition rate, evolving out of the thermal re-evaporation process of the thin films and grain growth [15,35]. The obtained dielectric constant for CdSe thin films grown at 150 °C and 300 °C had a peak value of 8, which is consistent with the literature [31]. However, the dielectric constant for the CdSe layer grown at 250 °C increased to 13, we believe, due to the observed crystal grain size increase (see Figure 2d). The dielectric constant of 5.3 at 400 °C was consistent with the change in crystalline orientation, as observed in XRD. The consistency of our results with those reported in the literature suggests that our CdSe films deposited by PLD had acceptable flatness, crystal structure, and composition.

## 4. Conclusions

CdSe thin films were deposited using the pulsed laser deposition method on ITO/glass substrate in a temperature range from 150 °C to 400 °C. The XRD spectra showed the presence of monocrystalline hexagonal CdSe, whereas the thin films grain size strongly was affected by the grain boundaries and was found to increase non-monotonically with the rise in deposition temperatures. The CdSe films surface morphology using AFM confirmed that the domain size was between 14 nm and 18 nm, and was much larger than the expected excitonic Bohr radius for CdSe (5.4 nm), which eliminates the occurrence of a quantum confinement effect. However, the possible of existence of smaller CdSe particles cannot be completely ignored at present. The CdSe optical band gap of ~1.71 eV, calculated using the Tauc plot method, matches with the spectroscopic ellipsometry results; however, for CdSe samples deposited at higher temperatures, a red shift of 0.1 eV was observed in the optical absorption edge. We observed that the maximum refractive index and low extinction coefficient for the CdSe samples occurred in the near band edge energy region due to the modification of the excitonic emission peaks at the absorption edge, and strongly affected by the CdSe thin film morphology. Interestingly, the CdSe sample grown at 400 °C showed a reduced PLD deposition rate and decrease in the thickness of CdSe thin film, resulting in XRD pattern change with respect to the crystal orientation of the hexagonal lattice structure. This observation was in agreement with the ellipsometry spectra and the AFM surface analysis. Such morphology change was evidenced further by the absence of well-defined absorption edge peaks observed for CdSe samples deposited at lower temperatures, and indicates that the substrate temperature at which CdSe thin films can be successfully deposited by PLD for photovoltaics applications should not exceed ~350 °C.

## Figures and Tables

**Table 1:** CdSe thin film roughness estimation from atomic force microscopy analysis.

Growth Temperature (°C)	150	200	250	300	350	400
RMS roughness (nm)	2.1	2.6	2.5	2.7	3.0	5.7

**Table 2:** Parameters extracted from spectroscopic ellipsometry.

Parameter/Temperature	150 °C	250 °C	300 °C	400 °C
Thickness (nm) of CdSe layer	184	253	241	163
*E_g_* (eV)	1.63 ± 0.03	1.71 ± 0.03	1.71 ± 0.03	1.67 ± 0.03
Refractive index *n_max_* (at 1.75 eV)	3.0 ± 0.2	3.6 ± 0.4	3.0 ± 0.2	2.4 ± 0.1
Extinction coefficient *κ_max_* (at 4.5 eV)	1.50	1.25	1.50	1.40
*ε_r_* (at 1.75 eV)	8.9	13.0	8.9	5.3
*ε_i_* (at 4.25 eV)	6.0	7.4	6.0	5.7
*α* (cm^−1^) (at 4.5 eV)	65 × 10^4^	70 × 10^4^	70 × 10^4^	65 × 10^4^

## Data Availability

The data presented in this study are available on request from the corresponding author.
